# NMR Investigation of the Supramolecular Complex Formed by a Phenylboronic Acid-Ferrocene Electroactive Probe and Native or Derivatized β-Cyclodextrin

**DOI:** 10.3390/ijms23116045

**Published:** 2022-05-27

**Authors:** Andrea Cesari, Maria Antonietta Casulli, Takeshi Hashimoto, Takashi Hayashita

**Affiliations:** 1 Department of Chemical Sciences, University of Padova, 35131 Padova, Italy; 2 Department of Materials and Life Sciences, Sophia University, Tokyo 102-8554, Japan; t-hasimo@sophia.ac.jp (T.H.); ta-hayas@sophia.ac.jp (T.H.)

**Keywords:** NMR spectroscopy, derivatized cyclodextrin, derivatized ferrocene, phenylboronic acid, dipicolylamine, electrochemical sensing, metabolites

## Abstract

Specifically designed electrochemical sensors are standing out as alternatives to enzyme-based biosensors for the sensing of metabolites. In our previous works, we developed a new electrochemical assay based on cyclodextrin supramolecular complexes. A ferrocene moiety (Fc) was chemically modified by phenylboronic acid (4-Fc-PB) and combined with two different kinds of cyclodextrins (CDs): β-CD and β-CD modified by a dipicolylamine group (dpa-*p*-HB-β-CDs) for the sensing of fructose and adenosine-triphosphate (ATP), respectively. The aim of the present work is to better comprehend the features underlining the aforementioned complex formation. For the first time, a study about inclusion phenomena between the 4-Fc-PB electroactive probe with β-CD and with dpa-*p*-HB-β-CD was performed by using nuclear magnetic resonance (NMR) analysis. In particular, we focused on providing insights on the interaction involved and on the calculation of the binding constant of 4-Fc-PB/β-CD supramolecular complex, and elucidation about a drift in the time observed during the control experiments of the electrochemical measurements for the 4-Fc-PB/dpa-*p*-HB-β-CD supramolecular complex. In this sense, this paper represents a step further in the explanation of the electrochemical results obtained, pointing out the nature of the interactions present both in the formation of the inclusions and in the sensing with the analytes.

## 1. Introduction

Metabolites are intermediate or final products of a metabolic pathway [1]. Their sensing is of great interest for the food industry to characterize nutritional aspects [2], whereas in medicine, metabolites are indicators of the human status of health, providing information about possible diseases or the monitoring of therapy [3,4]. Actually, the real popularization of electrochemical sensors is standing out from the other sensing methods (e.g., mass spectrometry [5] and optical techniques [6,7]). In particular, electrochemical enzyme-based biosensors showed great potentialities for the sensing of metabolites due to their high selectivity for the presence of an active site, and their capability to accommodate just one (in the case of oxidases [8,9,10]) or a family (in the case of cytochromes [11,12]) of metabolites. However, enzymes cause a series of problems, e.g., interference by ascorbic and uric acids, two molecules greatly present in the human body and that oxidate at the same potential of oxidases [13]. Moreover, there are not matching enzymes for all the metabolites of interest, as in the case of adenosine triphosphate (ATP).

In our previous works, we proposed a new electrochemical assay for the sensing of metabolites based on different supramolecular cyclodextrin complexes [14]. We synthesized a new type of ferrocene (Fc) electroactive probe modified by the phenylboronic acid group (4-Fc-PB), and we combined it with two different cyclodextrins: natural β-cyclodextrin (β-CD) for the sensing of fructose [15], and β-CD modified by a dipicolylamine group (dpa-*p*-HB-β-CDs) for the sensing of ATP [16]. In such a way, the different supramolecular complexes possess specific binding groups that act as lariats, grasping the desired target-molecule near the microenvironment constituted by probe/CD over the course of the electrochemical measurement and guaranteeing the sensing of the metabolite with very high selectivity.

There have essentially been two requirements for the choice of ferrocene as an electroactive probe for the electrochemical sensing of metabolites: the need for a molecule that is easily included in the cyclodextrins cavity and at the same time a molecule that is easy to chemically modify by specific binding groups (i.e., phenylboronic acid) for the interaction with the metabolites. In this sense, the ferrocene moiety has been shown to satisfy both of these requirements.

In order to obtain a detailed description on the origin of a receptor selectivity at a molecular level, Nuclear Magnetic Resonance (NMR) spectroscopy represents a powerful and faceted tool that is particularly useful for the deep characterization of cyclodextrins inclusion complexes [17,18], formed with both hydrophilic and lipophilic target molecules [19,20,21,22].

Herein, we report for the first time a study about the inclusion phenomena between the 4-Fc-PB electroactive probe with β-CD and with dpa-*p*-HB-β-CDs by using different NMR analyses, to shed light on the stereochemical features underlining the formation of electroactive probe/cyclodextrin supramolecular complexes. In particular, the paper focuses on the calculation of the binding constant of the 4-Fc-PB/β-CD supramolecular complex, on the host/guest interactions involved, and on the elucidation about the origin of a drift in the time observed during the control experiments of the electrochemical measurements for the 4-Fc-PB/dpa-*p*-HB-β-CD supramolecular complex.

## 2. Results and Discussion

### 2.1. Characterization of the Binding Partners

**Figure 1:**
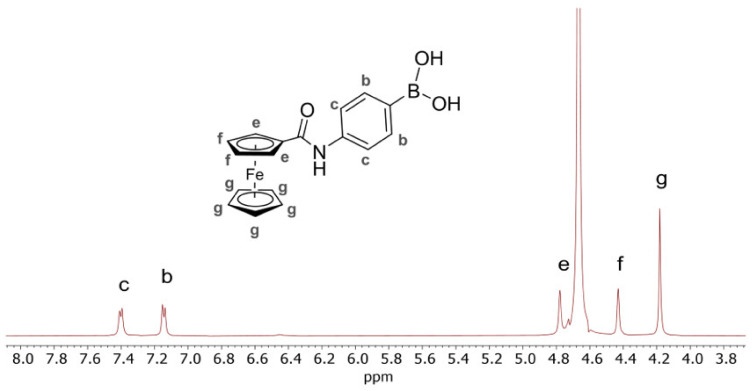
^1^H NMR (500 MHz, 5 mM, DMSO-d_6_/D_2_O 1:10 v/v, and [Na_2_CO_3_] = 10 mM) spectrum of 4-Fc-PB.

Before investigating the 4-Fc-PB/CDs interaction, a complete characterization of both derivatized ferrocene (4-Fc-PB) and derivatized 3-NH_2_-β-CD (dpa-*p*-HB-β-CD) was a necessary step. In the ^1^H NMR spectrum of 4-Fc-PB, the aromatic protons of the phenyl-boronic acid were easily recognized at 7.43 ppm (H_c_) and 7.18 ppm (H_b_), whereas the protons belonging to the ferrocene derivatized ring were distinguished in the spectral region 4.0-5.0 ppm (Figure 1, H_e_ 4.78 ppm, H_f_ 4.43, and H_g_ 4.18 ppm).

**Figure 2:**
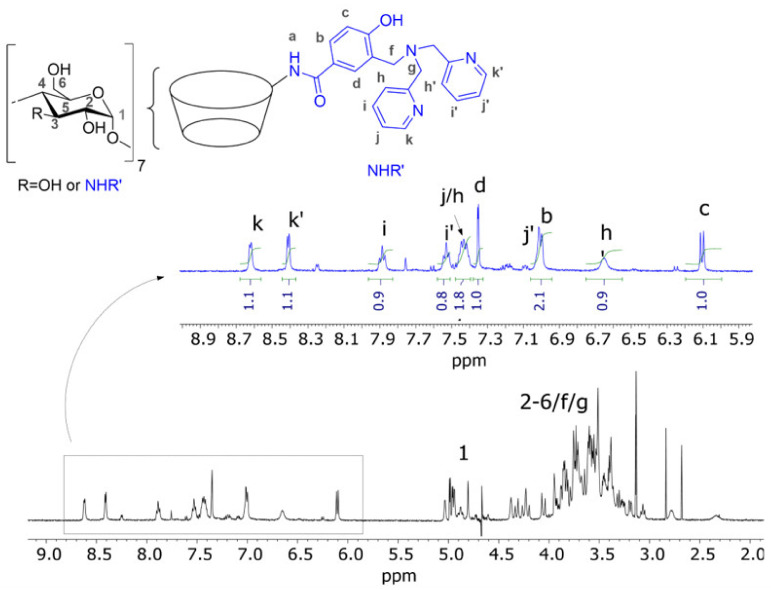
The ^1^H NMR (500 MHz, 25 °C, D_2_O) spectrum of dpa-*p*-HB-β-CD.

In the case of dpa-*p*-HB-β-CD, the signals of its covalently linked aromatic moiety were fully assigned by using ^1^H NMR spectrum analysis (Figure 2) and 2D COSY map (Figure S1). The anomeric protons of dpa-*p*-HB-β-CD (H_1_) produced a cluster of different peaks at 4.7–5.1 ppm, indicating effective derivatization. Moreover, the comparison between the proton unit of the dpa-*p*-HB-β-CD aromatic ligand (1H, ~1.0) and the integrated area of the anomeric protons (7H, ~6.8) agree well with a single derivatization on C_3_-NH_2_, as expected. Respect to pure β-CD, in which its symmetrical toroidal geometry produces a unique and well resolved set of signals, derivatized dpa-*p*-HB-β-CD showed the existence of conformational distortions and an unsymmetrical chemical environment. As a consequence, the derivatization produced a complicated ensemble of splitting and overlapping signals in the region of dpa-*p*-HB-β-CD ring protons (H_2_–H_6_), which made the task of obtaining information on the stereochemistry of 4-Fc-PB/dpa-*p*-HB-β-CD inclusion difficult.

### 2.2. ^1^H NMR Investigations on 4-Fc-PB/β-CDs Supramolecular Complex

**Figure 3:**
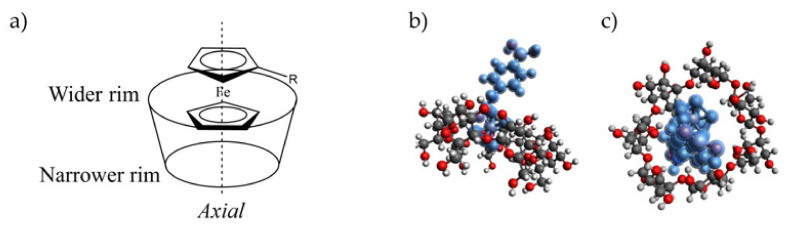
(**a**) The axial orientation of derivatized ferrocene inside a cyclodextrin cavity; (**b**) the side and (**c**) top view of a 3D molecular representation of the 4-Fc-PB probe in a natural β-CDs cavity.

The calculation of the binding constant of a ferrocene (Fc) probe interacting with a CD cavity is challenging enough. One of the most typically used techniques for the determination of binding constant is UV-Visible spectroscopy [23]. However, the spectrophotometric method is difficult to apply to the case of Fc since this molecule presents very small spectral variations [24]. Attempts to evaluate the Fc/β-CD binding constant have been determined as 2.2 × 10^3^ M^−1^ using molecular dynamic calculations [25,26,27], while solubility measurements based on the vapor-circulation technique have calculated the association constant of Fc with β-CD as 1.7 × 10^4^ M^−1^ [28] in water. In this context, it is also commonly accepted that ferrocene prefers an axial orientation over an orthogonal orientation for its inclusion inside the β-CD cavity (Figure 3) [25].

The first step of our investigation was firstly to verify the inclusion of the 4-Fc-PB probe in natural β-CDs and secondarily to evaluate the correspondent binding constant by ^1^H NMR studies. Such an analysis turned out to be necessary in our case since we hypothesized that the phenylboronic group added to the Fc moiety causes a stoichiometric bulk, reducing the binding constant of the inclusion reported in the literature.

A preliminary titration was carried out to exclude any self-association phenomena of 4-Fc-PB within the concentration range used for the association constant determination (0.1–10 mM), which could generate spurious chemical shift variations. As a matter of fact, no shift of 4-Fc-PB proton peaks was observed over 3 orders of magnitude of concentration variations (Figure S2); therefore, it was possible to solely attribute variation in the chemicals shifts of the 4-Fc-PB probe to the interaction with β-CD. For that, ^1^H NMR titration was carried out in the presence of a fixed concentration of β-CD (5 mM), and chemical shifts of different proton peaks related to both ferrocene and β-CD were collected (Figures S3 and S4). The proton shifts of both the H_g_ proton of the 4-Fc-PB probe and the H_3_ protons of the internal cavity of natural β-CDs confirmed the inclusion. Moreover, by fitting the chemical shift variations using a 1:1 model (Figure S5), it was possible to estimate the association constant as *K_a_* = 385 ± 9 M^−1^ using H_g_ protons and *K_a_* = 765 ± 8 M^−1^ using H_3_^CD^, with a sufficient accordance between the two values (and both were inferior to Fc/β-CD K_a_ ≈ 10^3^ M^−1^ [25,26,27]). This fact can be explained due to the presence of the phenylboronic acid group, which creates additional steric hindrance.

In the case of 4-Fc-PB, a phenylboronic acid moiety is conjugated to one pentadienyl ring of Fc. Since β-CD possesses a hydrophobic cavity capable of accommodating the aromatic portions of a wide variety of compounds, another important point to investigate was the eventual inclusion of the PB moiety into β-CD. In this regard, it is noteworthy that the signals of the protons H_c_ and H_d_ of 4-Fc-PB did not change over the titration in the presence of β-CD, hinting that the phenylboronic acid portion is likely not involved in the interaction.

**Figure 4:**
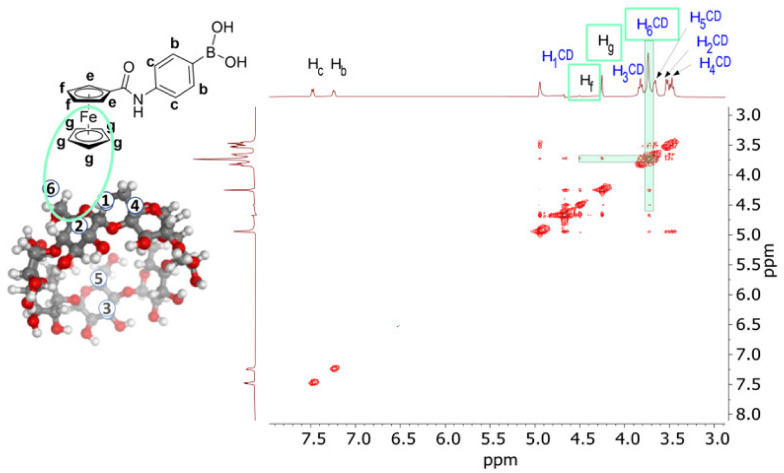
A 2D NOESY map (500 MHz, DMSO-d_6_/D_2_O 1:10 v/v, [Na_2_CO_3_] = 10 mM, mix = 0.2 s, 25 °C) of [4-Fc-PB probe] = 10 mM and [natural β-CDs] = 10 mM.

To gain deeper insight into the stereochemistry of the inclusion, a 2D NOESY map has been recorded and carefully analysed (Figure 4). The non-involvement of the PB fragment in the β-CD inclusion was confirmed once again since no cross-peaks were detected between the protons H_b_/H_c_ and any of H_n_^β-CD^. This result clearly indicates that the sensing fragment of 4-Fc-PB remained free to exert its activity during the electrochemical analysis. Conversely, NOE effects were detected between H_f_/H_g_ and H_6/6′_^β-CD^ protons, indicating an interaction between the ferrocenyl moiety of 4-Fc-PB and the lower rim of CD, with a stronger NOE effect between H_g_ and H_6/6′_^β-CD^ with respect to H_f_ and H_6/6′_^β-CD^. This is in line with the most sensible 4-Fc-PB protons observed along the NMR titration. However, in the specific case of medium-sized complexes (~1–2 kDa), weak or even null NOE values are due to an unfavourable correlation time (ωτ_c_ ~1, where ω is the Larmor angular frequency and τ_c_ is the molecular rotational correlation time of the complex) [29]. To exclude any undetected cross-peaks due to this eventuality (MW of 4-Fc-PB + β-CD = 1.48 kDa), a 2D ROESY map of the same 4-Fc-PB/β-CD mixture was also made, and both 2D NOESY and ROESY showed the same results (Figure 4 vs. Figure S6).

### 2.3. Characterization of 4-Fc-PB/dpa-p-HB-β-CDs Supramolecular Complex

**Figure 5:**
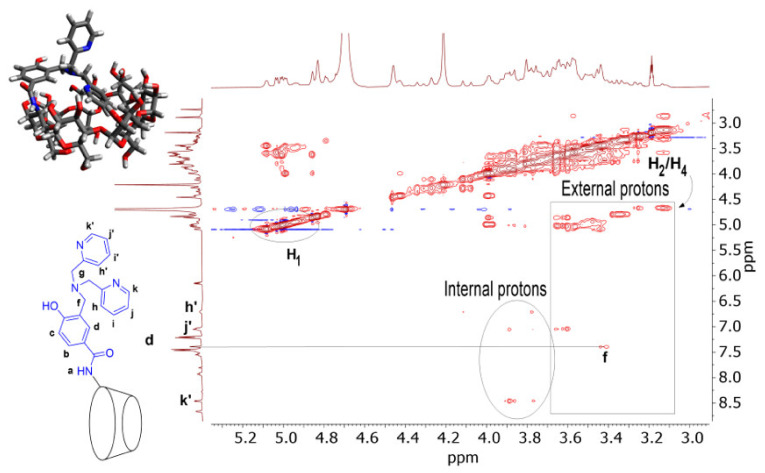
A 2D NOESY map (500 MHz, DMSO-d_6_/D_2_O 1:10 v/v, [Na_2_CO_3_] = 10 mM, mix = 0.5 s) spectra of [4-Fc-PB] = 5 mM + [Zn^2+^/dpa-*p*-HB-β-CD] = 5 mM mixture.

The description of 4-Fc-PB/β-CD helped in the better rationalization of the more complicated 4-Fc-PB/dpa-*p*-HB-β-CD system. As demonstrated in ref. [16], the presence of the dipicolylamine group is of primary importance for the sensing of ATP since it entraps Zn^2+^ ion, creating an electrostatic bonding with the three phosphate groups of ATP. However, the complex 4-Fc-PB/dpa-*p*-HB-β-CD developed a time drift during the performance of the control experiments [16] (Figure S7), on the contrary to the case of 4-Fc-PB/β-CD used for the sensing of fructose [15] (Figure S8). The origin of the time drift was identified in the possibility of intramolecular self-inclusion due to the different degrees of freedom of movement of its lateral arm since dpa-*p*-HB-β-CD is derivatized with a dipicolylamine group. Actually, such a phenomenon was effectively hypothesized by a 3D modelling (Figure S9). A 2D NOESY map confirmed the hypothesis, highlighting the interaction between protons H_h’_, H_j’,_ and H_k’_ belonging to the outer pyridinyl ring with the internal protons H_3_^β-CD^ and H_5_^β-CD^ of the cyclodextrin cavity (Figure 5). In principle, the occurrence of the combination of intra- and intermolecular inclusions is not to be excluded.

It is reasonable to think that the 4-Fc-PB probe inclusion is not stable over time because the CD cavity is partially occupied by the dipicolylamine group. The limited inclusion of 4-Fc-PB inside dpa-*p*-HB-β-CD with respect to β-CD causes its faster degradation over time, resulting in the time drift observed.

The crowdedness of the 4-Fc-PB/dpa-*p*-HB-β-CD spectra and the effect arising from the self-association of the cyclodextrin did not allow one to evaluate the association constant by ^1^H NMR titration. However, the complexation shifts (Δδ_CS_ = δ_mixture_−δ_pure_, Hz) analysis of the 4-Fc-PB/dpa-*p*-HB-β-CD mixture showed that the most affected protons of 4-Fc-PB were those belonging to the ferrocene moiety (H_f_/H_g_ Δδ_CS_ = −0.03 ppm vs. H_b_/H_c_ = 0.00–0.01 ppm, Figure S10). Protons belonging to the amino-boronic fragment remained almost unperturbed, suggesting that, in this case too, the inclusion occurs from the cyclopentadienyl rings, leaving the electroactive fragment almost free. Signals produced by the aromatic protons of CD underwent minor variations (δ_CS_ = 0.01–0.02 ppm).

Since the ultimate application of the 4-Fc-PB/dpa-*p*-HB-β-CDs supramolecular complex is in the sensing of ATP, the effect of the presence of ATP was also observed in the ^1^H NMR spectrum (Figure S11). The signal broadening of the two Fc aromatic doublets (H_b_ and H_c_) was detected only in the copresence of ATP and dpa-*p*-HB-β-CDs, hinting at a synergic interaction, most likely exerted through cis-*diol* bonding with the ribose part of ATP and mediated by the presence of the CD. This result supports the detection mechanism introduced in ref. [16], where specific groups (i.e., phenyl-boronic moiety) grasp the desired target molecule, while the role of the dipicolylamine group was elucidated by electrochemical experiments since, in the absence of the dipicolylamine group, the ATP was impossible to detect [16].

## 3. Materials and Methods

Ferrocene carboxylic acid and 3A-amino-3A-deoxy-(2AS, 3AS)-β-CD (NH_2_-β-CD) were purchased from the Tokyo Chemical Industry Co., Ltd. (Tokyo, Japan) and used without further purification. Natural β-CDs and sodium hydroxide were obtained as special-grade reagents from Fujifilm Wako Pure Chemical Industries Ltd. (Osaka, Japan). Deuterium oxide (D_2_O), methanol, sodium carbonate, and dimethylsulfoxide-d_6_ (DMSO-d_6_) were purchased from Kanto Chemical Co., Inc. (Tokyo, Japan). All other organic solvents, monosaccharides, and reagents were commercially available with guaranteed grade and used as received. Water was doubly distilled and deionized by a Milli-Q water system (WG222, Yamato Scientific Co., Ltd., Tokyo, Japan and Milli-Q Advantage A10, Burlington, MA, USA) before use.

Proton nuclear magnetic resonance (^1^H NMR), 2D COSY (homonuclear COrrelation SpectroscopY), Nuclear Overhauser Effect SpectroscopY (NOESY), and Rotating Frame Overhauser Enhancement SpectroscopY (ROESY) spectra were measured with a JNM-ECX500 (JEOL Ltd., Tokyo, Japan) at 300 K operating at 500 MHz for ^1^H. pH values were recorded using a Horiba F-52 pH meter (Horiba, Ltd., Kyoto, Japan). Differential pulse voltammetry (DPV) measurements were performed by Model 440A potentiostat by ALS CH Instruments Inc. (Austin, TX, USA) using an undivided three-electrode cell in Argon.

All the ^1^H NMR measurements were performed by diluting 4-Fc-PB and CDs in DMSO-d_6_/D_2_O (1:10 v/v), in the presence of Na_2_CO_3_ (10 mM), to simulate, as much as possible, the same conditions of the electrochemical experiments. For both the NOESY and ROESY studies, the sample has been prepared as follows: 5 mM of 4-Fc-PB probe and 5 mM of natural β-CDs (or dpa-*p*-HB-β-CDs) have been diluted in DMSO-d_6_/D_2_O (1:10 v/v), in the presence of Na_2_CO_3_ (10 mM), to simulate, as much as possible, the same conditions of the electrochemical experiments. NMR data processing for the determination of the association constant was carried out by DynaFit software ver. 4.09.012, using a 1:1 stoichiometry binding model [30]. 3D modelling was drawn using Avogadro 1.2.0.

## 4. Conclusions

Within this work, a detailed NMR investigation on inclusion phenomena between a 4-Fc-PB electroactive probe with β-CD and with dpa-*p*-HB-β-CD has been reported, shedding light on the stereochemical features of the complexes. In particular, the calculation of the binding constant of the 4-Fc-PB/β-CD supramolecular complex through ^1^H NMR titration analysis has been carried out. The association constant was inferior to the Fc/β-CD because the presence of the bulky phenylboronic acid group of 4-Fc-PB acts as hindrance added to the Fc moiety. Nonetheless, the 2D NMR studies (NOESY and ROESY) showed that the phenylboronic acid portion is not involved in the inclusion and thus is capable of exerting its electrochemical activity. It was possible to further elucidate the origin of a drift in the time observed during the control experiments of the electrochemical measurements for the 4-Fc-PB and dpa-*p*-HB-β-CD supramolecular complex. The 2D NOESY map confirmed that this drift is mainly caused by the self-inclusion of the dipicolylamine group in the CD cavity, hampering the proper inclusion of the electrochemical probe and exposing it to degradation over time. However, complexation shifts analysis of the 4 Fc-PB/dpa-*p*-HB-β-CD mixture showed that the most affected protons of 4 Fc-PB were those belonging to the ferrocene moiety, while those of the amino-boronic fragment remained almost unperturbed, suggesting that, in this case too, the inclusion occurs in a similar modality. Finally, the synergistic interaction of the phenylboronic moiety with ATP only in the co-presence of dpa-*p*-HB-β-CD has also been observed.

The obtained results are of primary importance for the future evaluation of other electroactive moieties as a valid alternative to the 4-Fc-PB probe. Promising applications are related to the chemical modification of other kinds of electroactive probes by the same specific binding groups discussed here (the phenylboronic acid and dipicolylamine groups). If the synthesis of these molecules will easily succeed, and by assuming that they will show a binding constant higher than the one reported for 4-Fc-PB probe in this work, we hypothesize that the electrochemical sensing of metabolites will be improved as consequence as well.

## Data Availability

Data is contained within the article or Supplementary Material.

## Supplementary Materials

The following supporting information can be downloaded at: https://www.mdpi.com/article/10.3390/ijms23116045/s1.
