# Supplementary material for: NMR Investigation of the Supramolecular Complex Formed by a Phenylboronic Acid-Ferrocene Electroactive Probe and Native or Derivatized β-Cyclodextrin
Source: Int J Mol Sci. 2022 May 27;23(11):6045. doi: 10.3390/ijms23116045 (PMC9181428; doi:10.3390/ijms23116045)
Supplement: Supplementary file 1 [file ijms-23-06045-s001.zip › supporting_information.pdf]

NMR investigation of the supramolecular complex formed by a phenylboronic acid-ferrocene electroactive probe and native or derivatized β-cyclodextrin

Andrea Cesari [1+*], Maria Antonietta Casulli [2+*], Takeshi Hashimoto [2], Takashi Hayashita [2]

1 Department of Chemical Sciences, University of Padova; andrea.cesari@unipd.it
2 Department of Materials and Life Sciences, Sophia University; m-casulli-5h6@sophia.ac.jp
* Correspondence: andrea.cesari@unipd.it (A. Cesari); m-casulli-5h6@sophia.ac.jp (M.A. Casulli)
+ These authors equally contributed to the work.

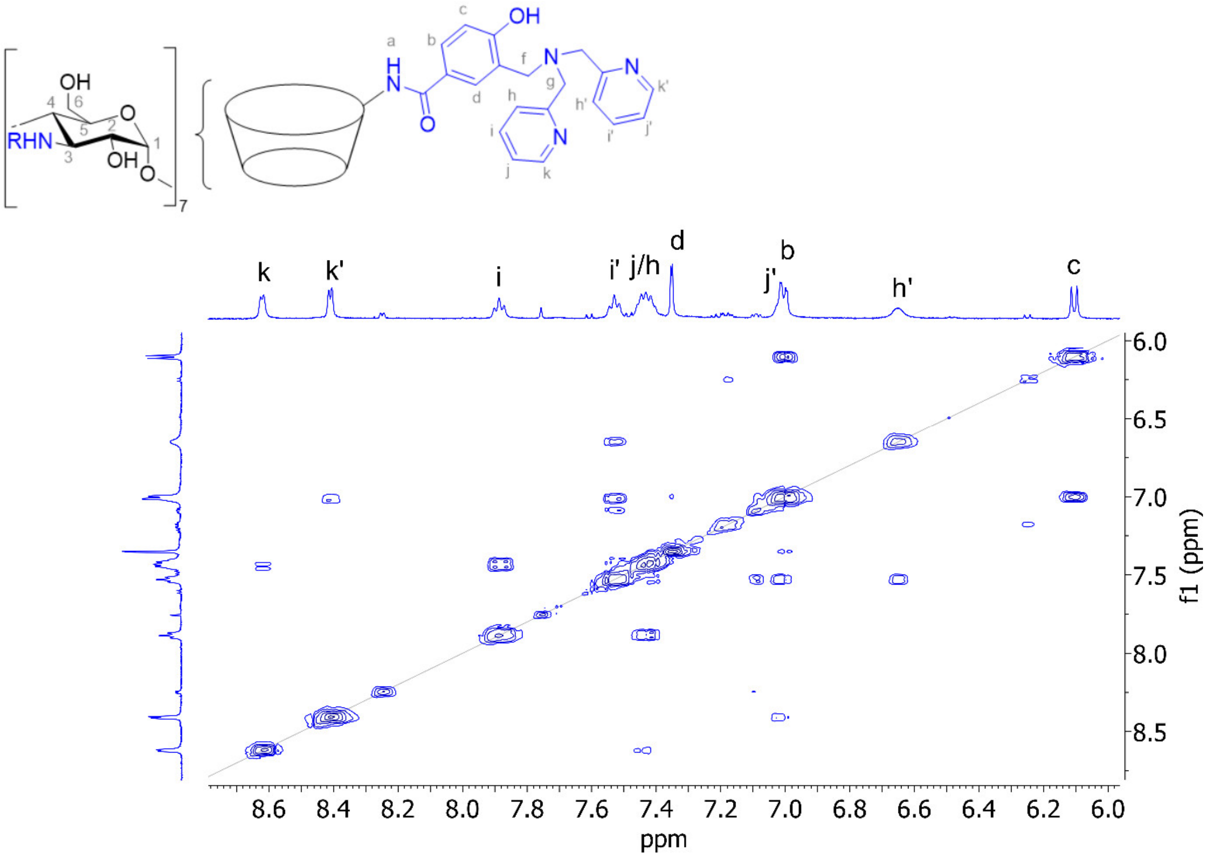

Figure S1. 2D COSY map (500 MHz, 25 °C, D_2O) spectrum of dpa-*p*-HB-β-CD.

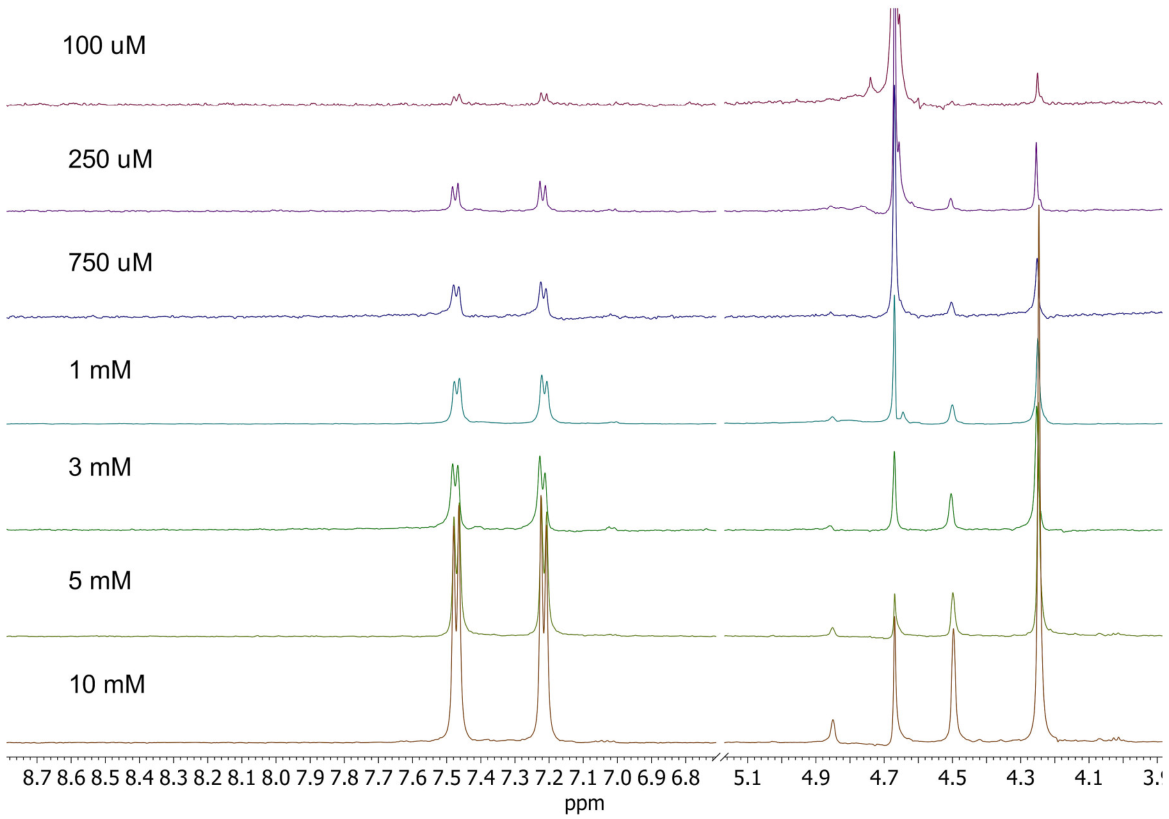

Figure S2. ^{1}H NMR titration (500 MHz. 25 °C, DMSO-d_6/D_2O (1:10 v/v), [Na_2CO_3] = 10 mM, [4-Fc-PB] probe = 0.1 mM ÷ 10 mM. No proton shifts of 4-Fc-PB have been observed, indicating the absence of any self-inclusion.

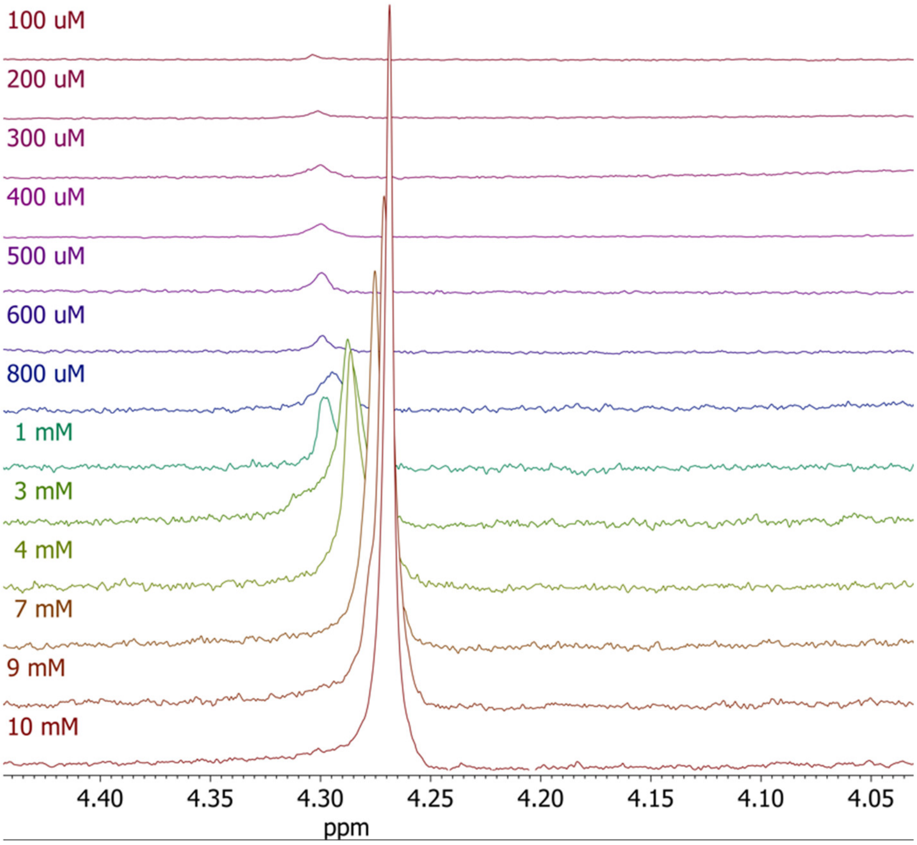

Figure S3. ^{1}H NMR titration (500 MHz. 25 ^{0}C, DMSO-d_6/D_2O (1:10 v/v), of [4-Fc-PB] probe = 0.1mM-10mM, [β-CDs] = 5 mM, [Na_2CO_3] =10 mM. Proton shift of H_g belonging to 4-Fc-PB have been observed indicating an inclusion in β-CDs.

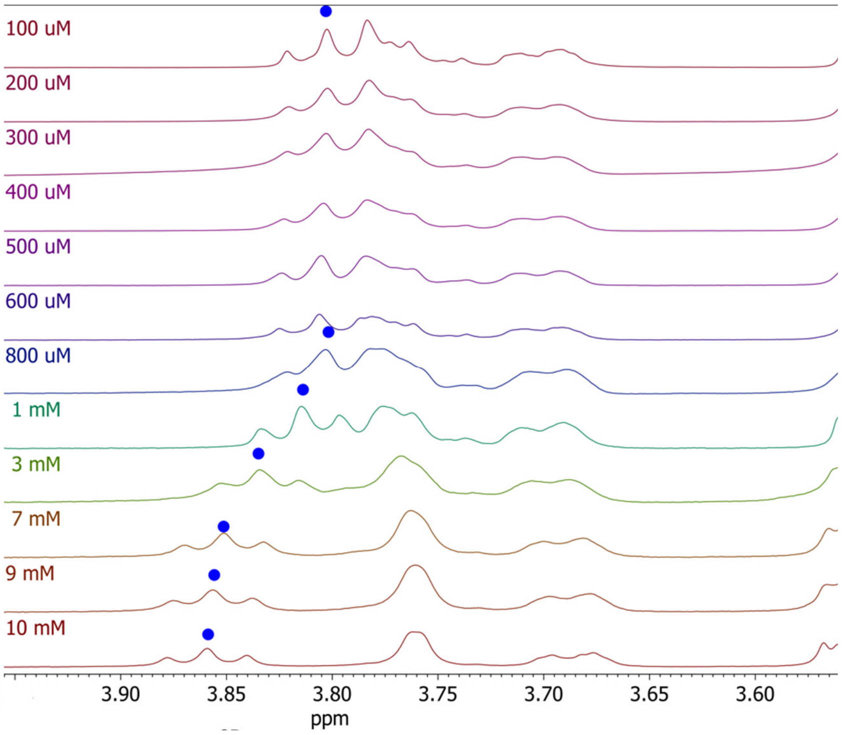

Figure S4. H NMR titration (500 MHz. 25 ^{0}C, DMSO-d_6/D_2O (1:10 v/v), of [4-Fc-PB] probe = 0.1mM-10mM, [β-CDs] = 5 mM, [Na_2CO_3] =10 mM. Proton shift of H_3 belonging to the internal

cavity of β-CDs have been observed indicating an inclusion of 4-Fc-PB probe in β-CDs.

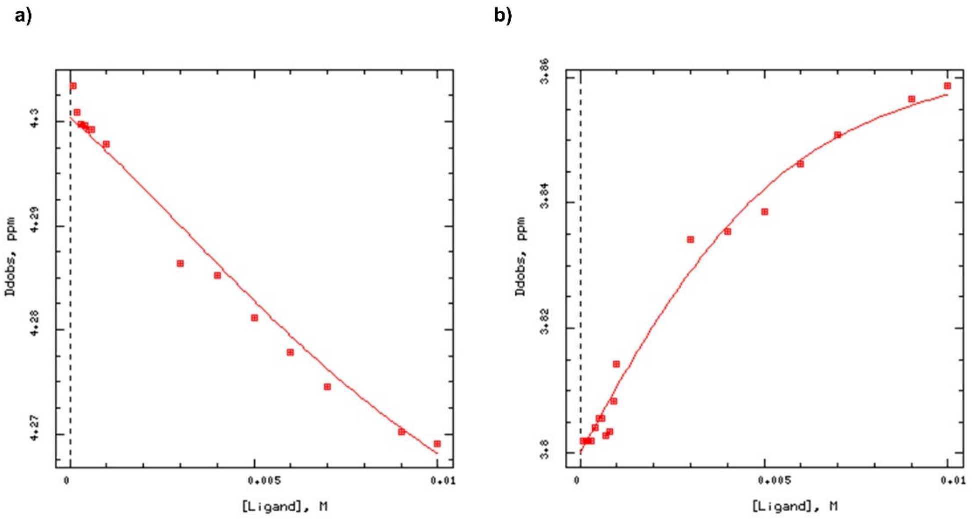

Figure S5. Plot of proton chemical shifts from ^{1}H NMR spectra, [4-Fc-PB] = 0.1 - 10 mM, [β-CDs] = 5 mM, in DMSO-d6/D2O (1:10 v/v), [Na_2CO_3] = 10 mM, 500 MHz. 25 ^{0}C. a) H_g protons shifts of 4-Fc-PB probe; b) H_3 protons shifts of natural β-CDs.

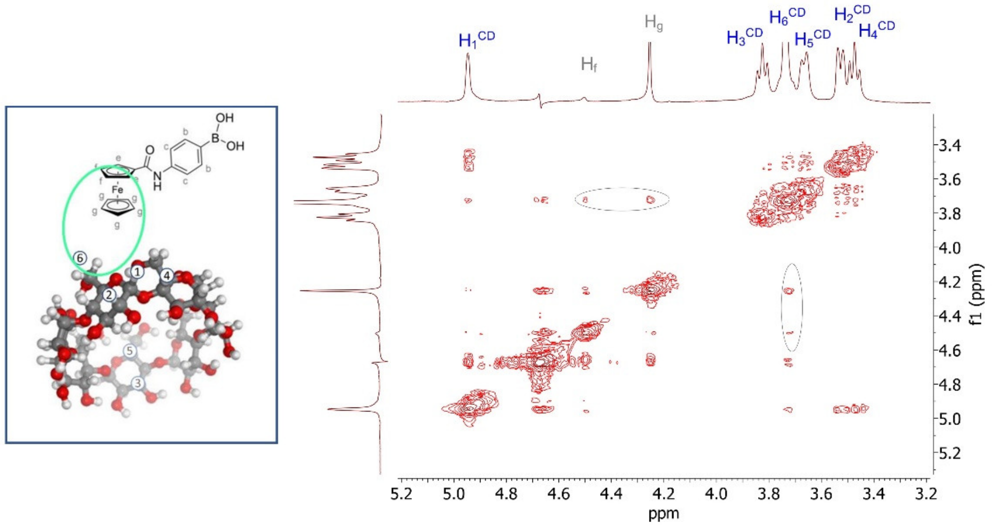

Figure S6. 2D ROESY map (500 MHz) of [4-Fc-PB] probe = 10 mM and natural [β-CDs] = 10 mM, DMSO-d6/D2O (1:10 v/v), [Na_2CO_3] = 10 mM, mixing time: 0.3 s; scans: 64; spinlock strength: 5.2 KHz.

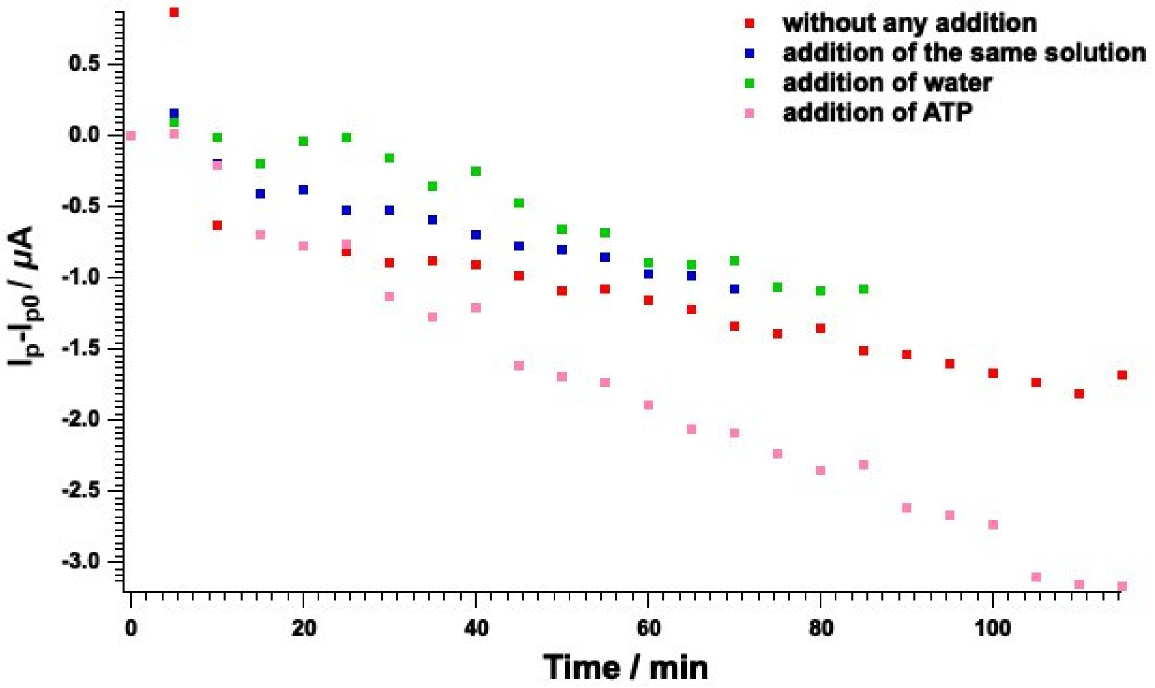

Figure S7. Trend in time (4-Fc-PB reduction peaks) by addition ATP (3 measurements for 0M, 200 μM, 300 μM, 400 μM, 500 μM, 600 μM, 750 μM, 1 mM for a total of 24 measurements) compared to three control experiments (addition of water, addition of the same solution of the electrochemical bath, no solution added). Measurements achieved in methanol: [$NaClO_4$] = 0.2 M (1:10 in v/v) water solution; [CHES buffer] = 1 mM (pH adjusted to 9.0 with NaOH).

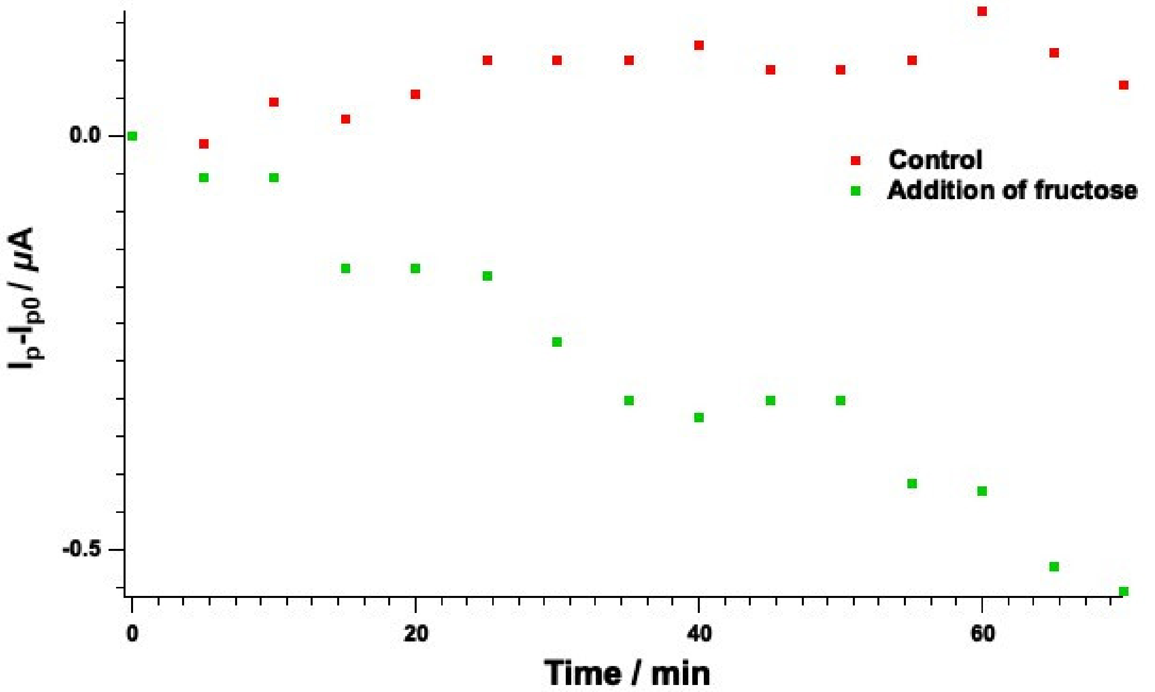

Figure S8. Trend in time (4-Fc-PB reduction peaks) by addition of fructose (3 measurements for 0M, 1 mM, 2 mM, 3 mM, 5 mM, for a total of 15 measurements) compared to the control experiment (addition of water). Measurements achieved in methanol: [$NaClO_4$] = 0.2 M (1:10 in v/v) water solution; [NaH_2PO_4 buffer] = 1 mM (pH adjusted to 9.0 with NaOH).

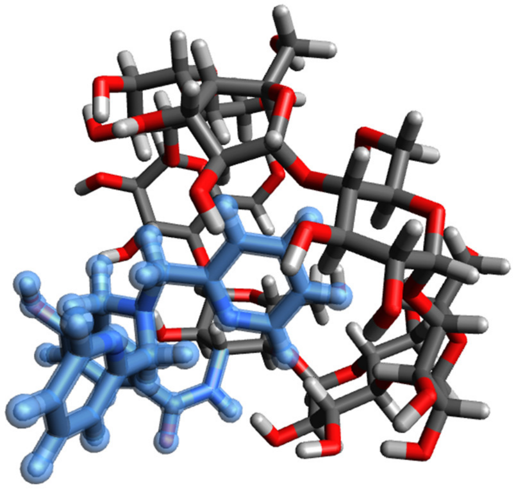

Figure S9. 3D representation of dpa-*p*-HB-β-CD, with dpa group highlighted in light blue colour.

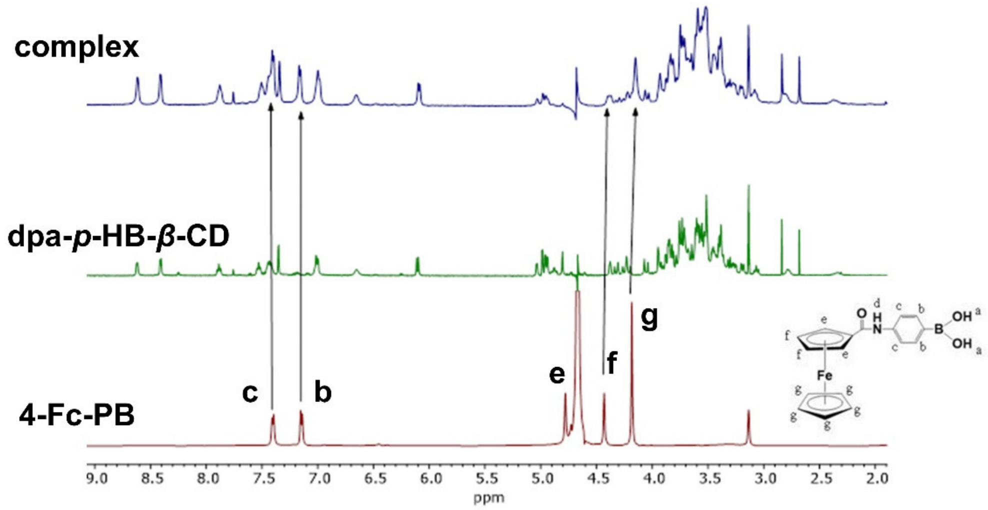

Figure S10. ^{1}H NMR (500 MHz, DMSO-d_6/D_2O (1:10 v/v), [Na_2CO_3] = 10 mM) spectrum of pure [4-Fc-PB] = 5 mM (low), pure [dpa-*p*-HB-β-CDs] = 5 mM (middle) and [4-Fc-PB] = 5 mM/[Zn^{2+}dpa-*p*-HB-β-CD] = 5 mM supramolecular complex (top).

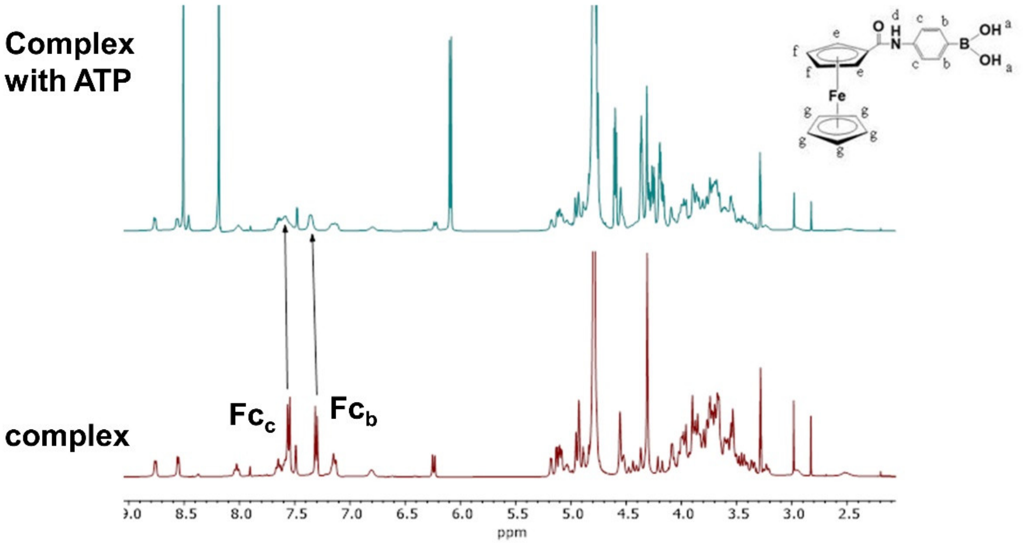

Figure S11. ^{1}H NMR (500 MHz, DMSO-d$_6$/D_2O (1:10 v/v), [Na_2CO_3] = 10 mM) spectrum of 4-Fc-PB/dpa-*p*-HB-β-CD supramolecular complex (5:5 mM) (low) and 4-Fc-PB/dpa-*p*-HB-β-CD/ATP (5:5:30 mM) (top).
